# Silver nanoparticles by atomic vapour deposition on an alcohol micro-jet

**DOI:** 10.1039/c9na00347a

**Published:** 2019-09-06

**Authors:** Michael J. McNally, Gediminas Galinis, Oliver Youle, Martin Petr, Robert Prucek, Libor Machala, Klaus von Haeften

**Affiliations:** Department of Physics and Astronomy, University of Leicester Leicester UK dr.michael.j.mcnally@gmail.com klaus.von.haeften@rub.de; Department of Engineering, University of Leicester Leicester UK; Regional Centre of Advanced Technologies and Materials (RCPTM), Faculty of Science, Palacký University Olomouc Olomouc Czech Republic; Kanano GmbH 89077 Ulm Germany

## Abstract

We achieved sputter deposition of silver atoms onto liquid alcohols by injection of solvents into vacuum *via* a liquid microjet. Mixing silver atoms into ethanol by this method produced metallic silver nanoparticles. These had a broad, log-normal size distribution, with median size between 3.3 ± 1.4 nm and 2.0 ± 0.7 nm, depending on experiment geometry; and a broad plasmon absorption band centred around 450 nm. We also deposited silver atoms into a solution of colloidal silica nanoparticles, generating silver-decorated silica particles with consistent decoration of almost one silver particle to each silica sphere. The silver–silica mixture showed increased colloidal stability and yield of silver, along with a narrowed size distribution and a narrower plasmon band blue-shifted to 410 nm. Significant methanol loss of 1.65 × 10^−7^ mol MeOH per g per s from the mature silver–silica solutions suggests we have reproduced known silica supported silver catalysts. The excellent distribution of silver on each silica sphere shows this technique has potential to improve the distribution of catalytically active particles in supported catalysts.

## Abstract 2

Nanoparticles produced when ethanol jet exits nozzle (a), over silver vapour source (b), captured in a cold trap (c).

## Graphical abstract

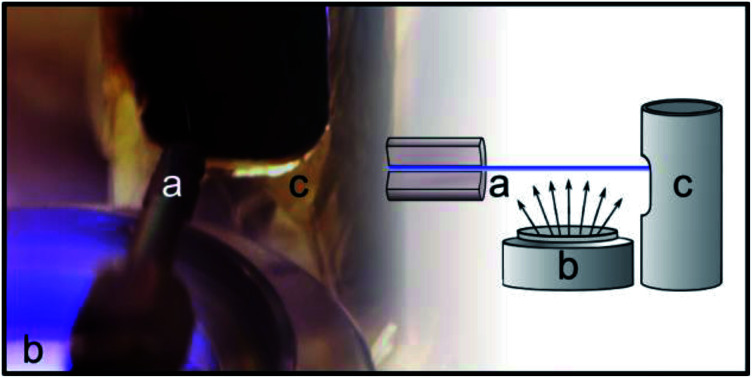

## 1 Introduction

Chemical synthesis of nanoparticles is a very broad field; with mature synthesis protocols for a wide range of particle chemistries, morphologies, shapes, sizes, stoichiometries, alloys and functionality; and sound empirical and theoretical understanding of the processes involved.^1–24^

Despite this success, there has been ongoing and intensifying work investigating “physical” methods of nanoparticle synthesis. Physical nanoparticle synthesis typically substitutes the chemical reduction of metal atoms or ions from precursor chemicals for a physical method. Early methods include the solvated metal atom dispersion (SMAD) method,^25–27^ whereby metal atoms are evaporated in a vacuum, along with a liquid vapour; both the metal atoms and liquid vapour co-deposit onto the liquid nitrogen cooled walls of the chamber; on melting the frozen mixture, nanoparticles are formed. The original motivation for SMAD experiments was to generate highly reactive slurries of reactive metals which would be difficult to achieve by other means;^25^ in general the process produces metal particles with un-terminated surfaces which are sufficiently reactive to readily undergo digestive ripening, whereby a polydisperse dispersion of metal particles can be refined into a monodisperse colloid without significant mass loss.^26–28^

Generating particles with such ‘naked’ un-terminated surfaces has provided an inspiration for substantial further research in this area; another consequence of the ‘naked’ surface is increased catalytic activity,^29^ with immediate commercial/industrial relevance.

Another physical synthesis technology is laser ablation in solution, where metal atoms and ions are generated by laser ablation of a metal target immersed in a solvent^30–32^ or by irradiation of particles already suspended in a liquid.^33,34^ Similarly, control over nanoparticle growth and morphology has been demonstrated by selective plasmon excitation by laser irradiation.^35^ Particles produced by laser ablation often show colloidal stability without capping agents or stabilisers.^30–34^

A large body of work also exists investigating the generation of metal atoms by plasma techniques. Recently, interest has been shown in generating nanoparticles by aggregation in atmospheric pressure plasmas^36^ and deposition into solvents;^37,38^ alternatively the plasma can be used as an ion source to drive chemical reactions in a liquid.^37,39–41^

Finally, a large body of work also exists on the sputter deposition of metals onto liquid surfaces. Metal atoms are generated by a DC or RF plasma ions impacting onto a metal surface, and in pressures below 10^−2^ mbar will produce predominantly neutral, single atoms.^42–44^ Both ionic liquid (IL) and sufficiently low vapour pressure solvent surfaces are stable in low vacuum and have been investigated as targets for metal deposition and nanoparticle formation.^45–54^ In general, the synthesis in mineral oils is predominantly governed by physical parameters of the plasma,^47–49^ whilst synthesis in ILs is significantly governed by the chemistry of the IL.^46,50,53,54^ In both systems, particles are commonly stable without added stabilisers or capping agents.^45–54^

In this work we extend on the technique of sputter deposition onto liquid surfaces by introducing liquid into vacuum in the form of a liquid microjet. Liquid micro-jets have been widely used as a tool for studying molecular processes in liquids under vacuum conditions,^55–59^ and narrow jets or surfaces of high vapour pressure liquids can be generated which are stable in vacuum over timescales of hours.^60^ This has allowed us to perform sputter deposition onto the surface of a high vapour pressure liquid.

The high vapour pressure corresponds inherently to a low surface energy, and also low viscosity (and so high diffusion of dissolved species); hence, sputtered material will easily penetrate into the liquid at low sputter voltages,^46^ and rapidly mix with dissolved material. This means that we can, in principle, create interactions between dissolved material in the jet and sputtered atoms, with minimal perturbation of the dissolved material compared to other physical methods of nanoparticle production.

We chose to investigate as close to pure metal–solvent mixtures as possible. For this reason, no stabilisers or capping agents were used. We investigated three methodologies of sputtering silver into liquid alcohols: sputtering only onto the surface of an ethanol jet (jet-only-deposition, JOD); sputtering onto the surface of an ethanol jet, but mainly into the frozen ‘slush’ of captured jet and condensed vapour (jet-co-deposition, JCD); and finally, deposition onto a jet and frozen solvent of a solution of silica nanospheres dissolved in methanol (JCD-SiO_*x*_), in order to investigate the interactions with dissolved material in the jet.

## 2 Method

We generated a continuous micro-jet of solvent with a diameter of 50 μm in vacuum, using a custom built assembly with a modified HPLC fitting holding a 50 μm inner diameter fused silica capillary. This source was connected to a reservoir, backed by a high-pressure Ar gas line, and mounted inside the vacuum chamber of an Edwards Auto-306 thin film coater. Before starting the experiment, the process liquids were filled into the reservoir. We used HPLC spectrophotometric grade ethanol (Sigma-Aldrich 459828) or commercial silica (LUDOX-HS30, Grace, 20 nm diameter, charge stabilised with a sodium counter-ion) dissolved in methanol (HPLC spectrophotometric grade, Sigma Aldrich 34860).

**Fig. 1:**
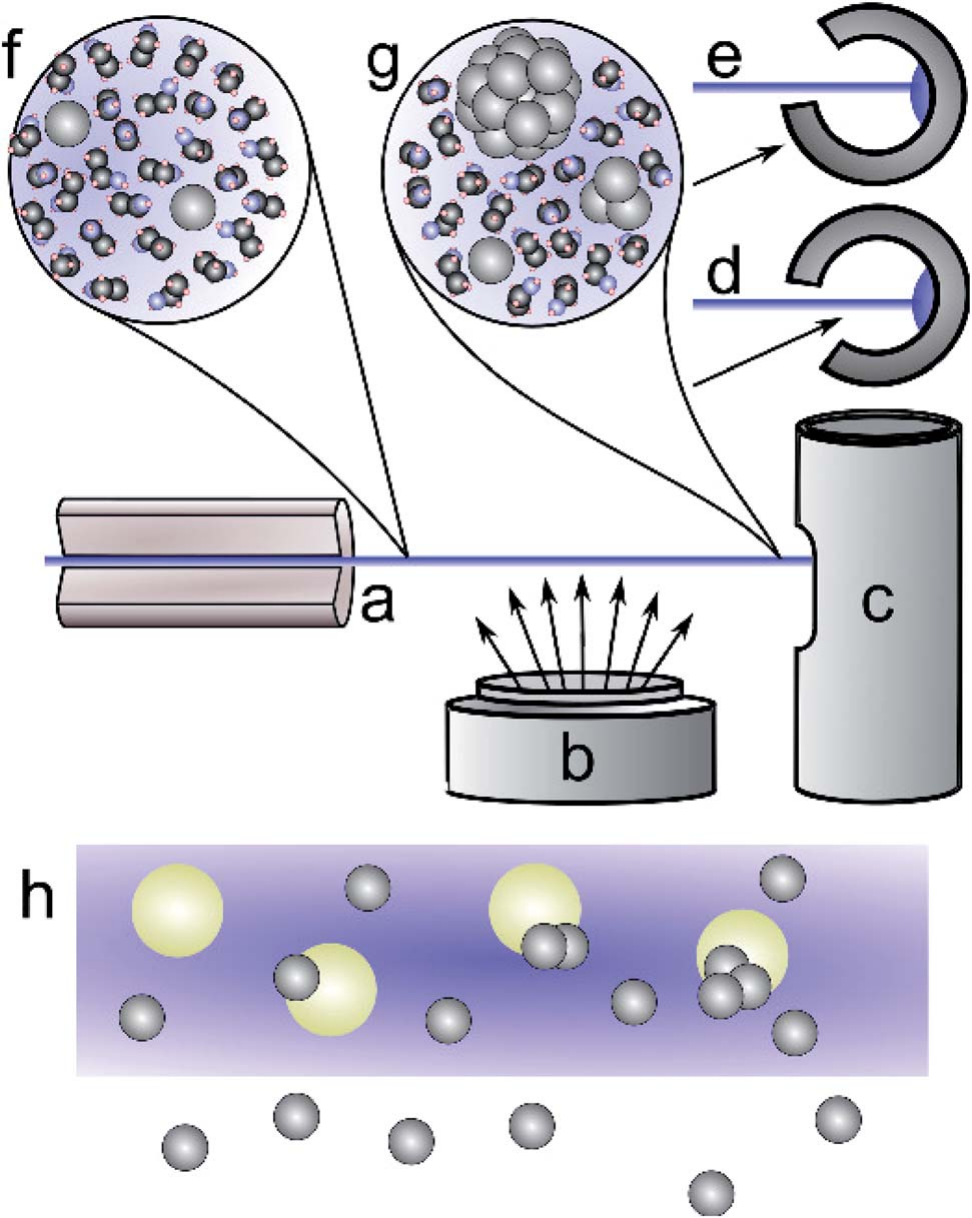
Schematic of the liquid jet sample production process. A narrow jet of ethanol is injected into vacuum through a 50 μm capillary (a), passes through atomic silver vapour produced by the sputter source (b), and is then frozen in a cold trap (c). The cold trap (plan view c, top view d and e) can be oriented to either allow silver to deposit with the frozen liquid (d), or to mask the interior (e), excluding direct deposition of silver atoms from the sputter source. Silver atoms reaching the liquid jet rapidly dissolve (f) and nucleate into small clusters (g) before reaching the cold trap (c). Deposition into a pure solvent jet (h) leads to silver–silver reaction to form small clusters. When silica particles are dissolved in the process solvent, the silver atoms can absorb and grow into particles on the silica surfaces.

A stainless steel cold trap was cooled in a bath of liquid nitrogen, transferred to the coater and oriented with its aperture for the chosen experiment (Fig. 1c–e). The coater vacuum chamber was evacuated to a pressure of 10^−6^ mbar, then argon (BOC Edwards ultra-pure) was introduced through a needle valve. At a chamber pressure of 5 × 10^−3^ mbar a DC magnetron discharge was ignited, generating an argon plasma at 580 V, 0.2 A, and sputtering silver atoms from a pure silver target (99.99%, Lesker EJTAGXX403A2). At the operating pressure this generated predominantly neutral silver atoms, with the expectation of producing a small percentage yield of silver ions^44^ and a maximum of 10% of 2–3 atom clusters.^43^ The deposition rate measured with a quartz crystal thickness monitor (Edwards FTM7) was 0.9 ± 0.1 Å s^−1^ at a distance of 25 cm from the centre of the sputtering target.

The measured deposition rate was used to estimate the concentration of silver in the sample, taking into account the relevant cross-sectional capture area. We calculated the sputter deposition rates at the liquid jet and at the cold trap by assuming an inverse-square relationship of sputtered material to radial distance of the sputter head. The cross section of the cold trap entrance, 38 mm by 30 mm, was taken as the area for sputtered silver collected in the cold trap during JCD experiments. The distance to the cold trap was 90 ± 0.5 mm. The cross section of the liquid jet was modelled as a trapezium, with sides of the jet diameter, 50 microns, and another of 1 mm to account for a jet divergence and fragmentation into droplets. We took an average deposition rate for distances between 50 mm and 90 mm to account for the liquid jet passing close to the sputter head. The schematic in Fig. 1 illustrates the scenario, where the liquid jet passes over the sputter head. Estimated concentrations are shown in Table 1.

**Table 1:** Comparison of concentrations, *c*. *c*_deposition_ is the estimated concentration loaded into the liquid sample during sample production. *c*_AAS_ is the concentration of all total Ag in samples aged for more than a year, measured by flame atomic absorption spectroscopy. *c*_plasmon_ is the estimated concentration of Ag atoms contributing to the plasmon resonances

Sample	*c*_deposition_	*c*_AAS_	*c*_plasmon_
JCD	166.1 mg L^−1^	7.6 mg L^−1^	11–15 mg L^−1^
JOD	10.9 mg L^−1^	4.6 mg L^−1^	3–14 mg L^−1^
JCD-SiO_*x*_	71.2 mg L^−1^	44.0 mg L^−1^	25–45 mg L^−1^

The liquid jet was established by rapidly raising the Ar backing pressure in the reservoir to 40 bar. Once the jet was established, we generated silver atoms by argon ion sputter evaporation at 5 × 10^−3^ mbar of Ar. The liquid jet will breakup rapidly into a stream of evenly spaced and sized droplets of diameter around 100 μm (ref. 61) but is otherwise stable. The jet undergoes minimal evaporation due to the small time it spends in vacuum, verified by the minimal increase in chamber pressure when the liquid jetting was started. Also, as there is no ambient atmospheric resistance, the droplets do not breakup any further. The experiments ran for approximately 5 minutes of deposition before the sputtering and jet were shut off. The chamber was then vented with argon and the frozen solvent was allowed to melt over roughly five minutes, which yielded typically several mL of colloid. Liquid flow rates through the capillary were calibrated by repeated measurements of exhausted volume in air and modelled by the Hagen–Poiseuille equation. At backing pressures of 40 bar, flow rates were 0.75 mL min^−1^ for ethanol and 1.75 mL min^−1^ for methanol. These correspond to droplet rates of 2.87 × 10^4^ droplets per s for ethanol and 6.69 × 10^4^ droplets per s for methanol.

Mixing metal atoms with solvent was explored by two different methods. Firstly, the cold trap was positioned so that sputtered material could reach the frozen solvent inside the trap, whilst also being captured in the liquid jet (Jet-Co-Deposition = JCD, Fig. 1d); secondly, by deposition onto the liquid jet only (Jet-Only-Deposition = JOD, Fig. 1e).^62^ No capping agents or stabilisers were added to the solvents in order to determining whether these methods would produce intrinsically stable nanoparticles, and whether any difference would be observed between the two regimes.

Images for survey samples (Fig. 3) were recorded by transmission electron microscopy (TEM) of dry films on ultra-thin carbon TEM grids in a JEOL 2100 microscope operated at 200 kV, under a range of magnifications. All samples used for TEM had been stored under ambient conditions without exposure to light for one year. Films were produced by drop-casting 2 μL colloidal solution of the undiluted sample onto the TEM grid (Agar scientific, S160). In order to acquire good quality TEM images of well spaced nanoparticles, JCD-SiO_*x*_ samples were diluted by a factor of 100 for drop casting.

**Fig. 2:**
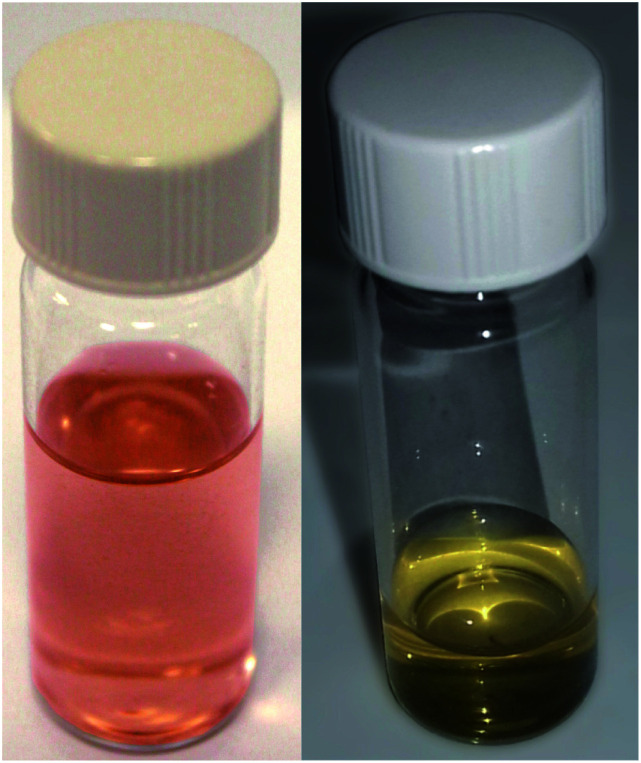
Sample photographs, left, sample produced by jet co-deposition. This is the colour that essentially all samples had immediately post production. Right, sample produced by jet co-deposition of a silica–methanol solution, after aging approximately three months.

**Fig. 3:**
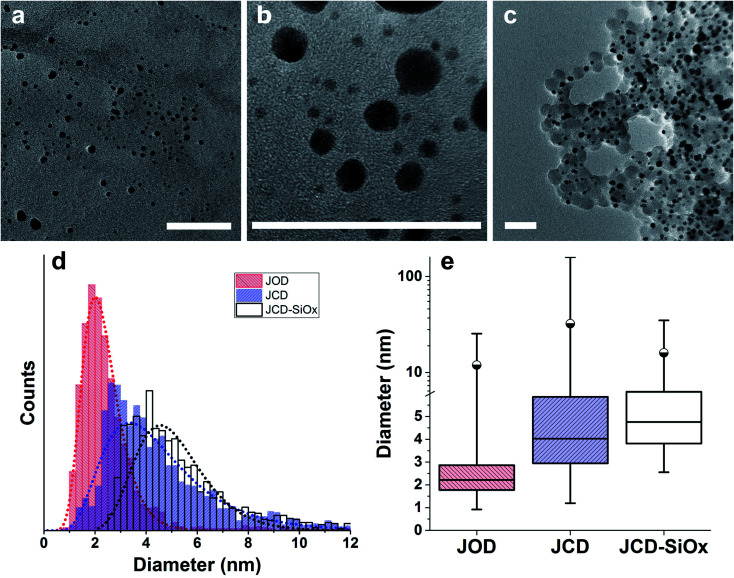
Transmission Electron Microscopy (TEM) survey images from sets used for size distributions. Scale bars are 50 nm. (a) Sample produced by Jet-Co-Deposition (JCD). (b) Jet-Only-Deposition (JOD). (c) Jet-co-deposition of silver into a solution of silica nanospheres (SiO_*x*_-JCD). The larger, lighter circles are silica particles, the smaller black particles are silver nanoparticles. (d) Size distribution of silver particles recorded from TEM survey images. The dotted lines are log-normal fits. (e) The same data plotted to show median (horizontal centre line), 25/75 percent limits (box), 99^th^ percentile (circle) and minimum/maximum limits (vertical line ends). The detection limit was 0.8 nm diameter for all JOD and JCD samples, whilst the detection limit for JCD-SiO_*x*_ samples was 1.4 nm.

Over 2000 nanoparticles were analysed using image processing software (ImageJ) to produce size distributions (Fig. 3d). Sizes below 0.8 nm could not be analysed as the image processing software could not distinguish individual particles from the background noise, although some could be distinguished by eye. Size distributions were fit with a log-normal distribution function, as defined in eqn (1) with mean *x̄* and multiplicative standard deviation *σ*. Errors quoted are the average differences between the 68% confidence intervals and the median, given by eqn (2).1
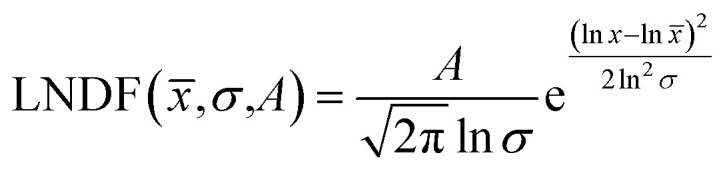
2
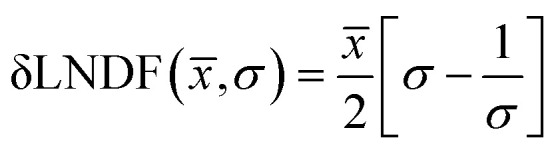


**Fig. 4:**
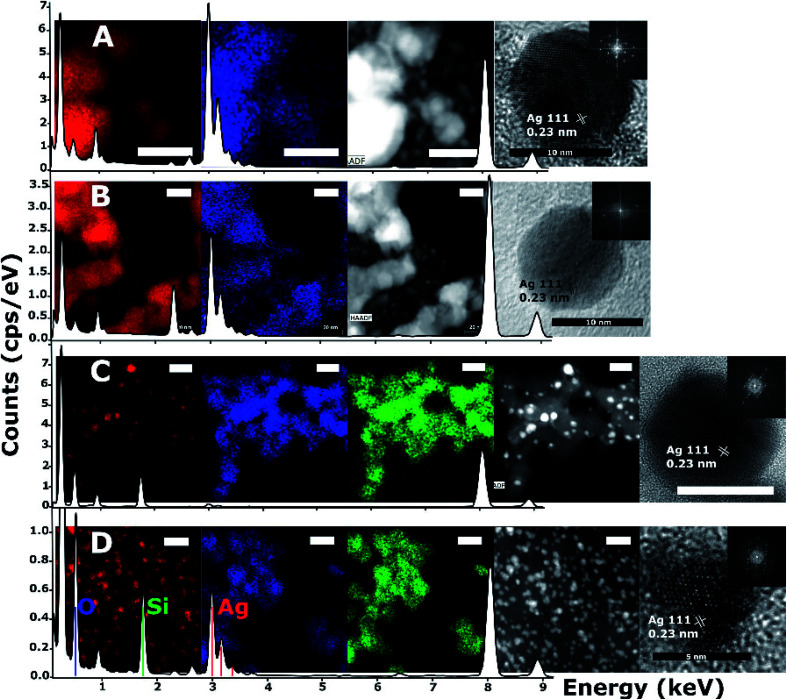
Energy dispersive and dark field microscopy of nanoparticle samples drop-cast onto lacey carbon supports, overlaid on EDS survey spectra of the pictured region. Samples of JCD (A), JOD (B) and JCD-SiO_*x*_ (0.01%), both the stable solution phase (C), and the solution when shaken (D). All scale bars are 20 nm unless labelled otherwise. From left to right, (A and B) silver EDS signal, oxygen EDS signal, dark field image, HRTEM of single nanoparticle and (inset) FFT. Similarly, (C and D) silver EDS signal, oxygen EDS signal, silicon EDS signal, dark field image, HRTEM of single nanoparticle and (inset) FFT. Unlabelled peaks in the EDS spectra are due to carbon and copper from the support. All measurements on HRTEM images are taken from FFT of individual particle images.

HRTEM images, high angle annular dark-field (HAADF) images and scanning EDS were acquired on an FEI Titan microscope. The survey area for the spectra shown in Fig. 4 corresponds to the regions pictured in the respective HAADF and EDS survey images, partially cropped to fit a square in some cases. Atomic planar spacing shown on the HRTEM images was measured from the FFT of the images and matched to known silver planar spacing and geometry in order to assign lattice planes.

**Fig. 5:**
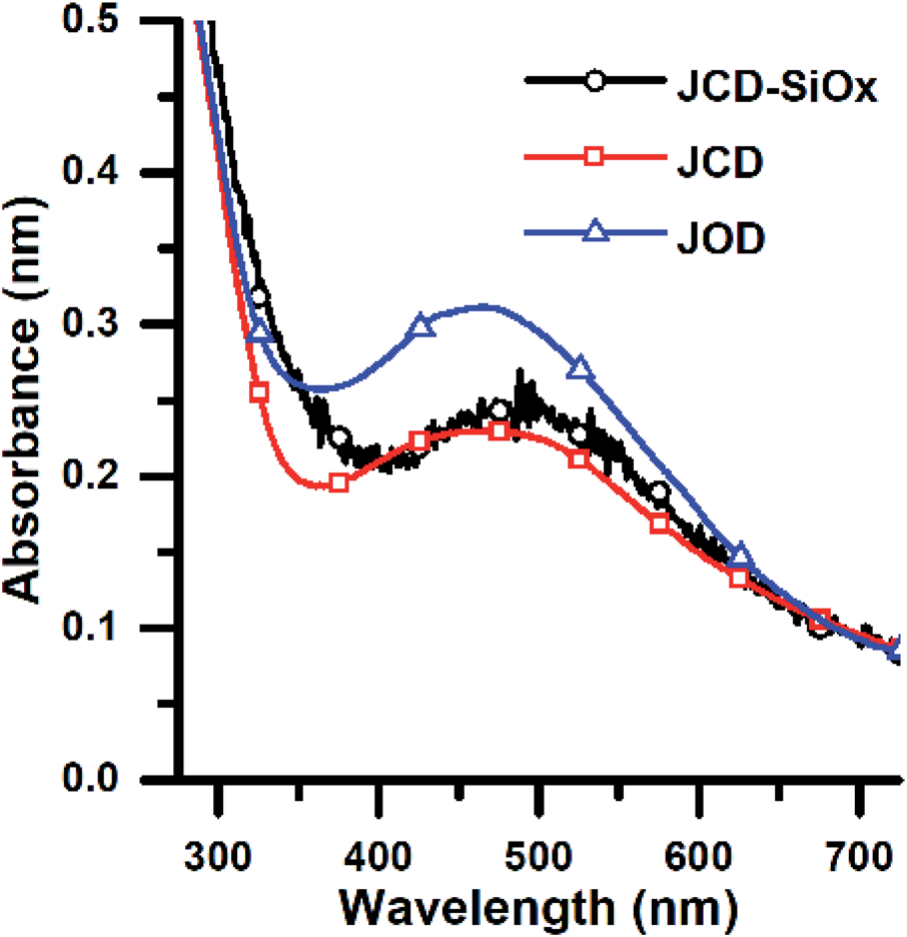
Optical absorption spectra of colloidal nanoparticle solutions of produced by Jet-Co-Deposition (JCD) of silver into ethanol, Jet-Only-Deposition (JOD) of silver into ethanol and by jet-co-deposition of silver into a solution of silica nanospheres (JCD-SiO_*x*_) in methanol. Spectra were recorded as produced (within 10 minutes), selected from samples with similar as-produced concentrations.

UV-vis spectra were recorded on a Thermo Evolution 220 spectrometer with a resolution of 0.5 nm. Samples were transferred to Suprasil quartz cuvettes from Hellma for measurements. Between sample measurements, samples were stored at 21 °C in the dark (Fig. 5).

**Fig. 6:**
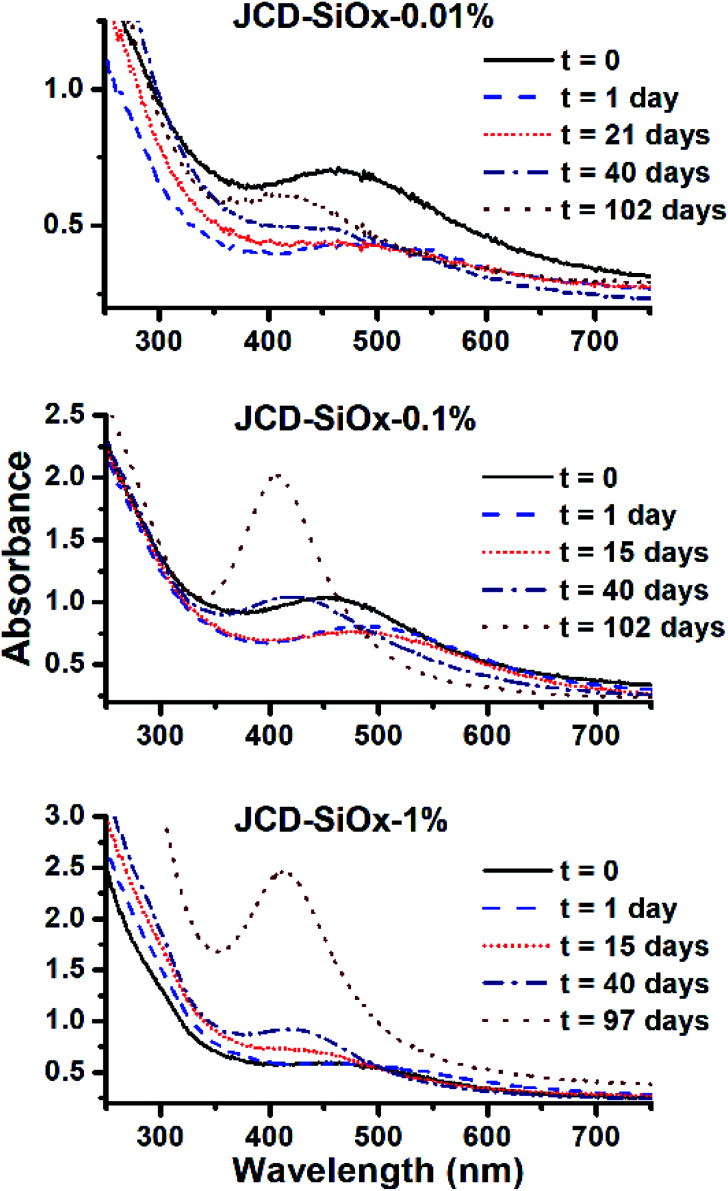
Evolution of the optical absorption spectra of JCD-SiO_*x*_ samples with different concentrations of silica nanoparticles. From top to bottom, concentrations of 0.01%, 0.1% and 1% LUDOX colloidal silica were added prior to jetting and sputtering silver.

To measure sample peak positions and absorbances, spectra had a flat baseline subtracted to equalise the height of a consistent 910 nm water vapour absorption. Peaks were fit by a Gaussian between the onset of the strong UV absorption edge (380 to 400 nm) and 600 nm. We used the method of Paramelle *et al.*^63^ for nanoparticle molar extinction coefficients of citrate-terminated spherical silver nanoparticles in water. Whilst our nanoparticles are unterminated, and in ethanol, there is good evidence that (unlike the peak position) the total absorbance of silver nanoparticles is not dramatically altered (*i.e.* not order of magnitude changes) by surface capping^64^ or particle shape.^65^ We converted the peak absorbances from low and broad peaks (*e.g.*Fig. 5) into low estimates of concentration. We took a high end estimate by converting the absorbance of a sharp peak from spectra illustrated in Fig. 6 into a concentration. The areas of the high and low peaks used were from the same sample and had areas matching to within 5%. This high estimate was then divided by the peak area, and multiplied by the area of a low, broad peak (particularly to produce a high estimate for samples which only showed broad absorption peaks). These two concentrations are quoted in Table 1. We feel that this method appropriately corrects for the polydispersity of our sample and accounts for the possible range of particle sizes and shapes.

Silver concentrations were determined by means of atomic absorption spectroscopy (AAS) with flame ionisation using a ContrAA 300 (Analytik Jena AG, Germany) equipped with a high-resolution Echelle double monochromator (spectral bandwidth of 2 pm at 200 nm) and with a continuum radiation source (xenon lamp). The absorption line used for these analyses was 328.0683 nm. Calibration standards of Ag for performing AAS were of TraceCERT (1 g L^−1^) type, purchased from Fluka.

## 3 Results and discussion

After sputtering silver into ethanol, by the JCD method, the melted solution had a pink colour (Fig. 2) which, despite some precipitation, retained its colouration for over one month. We could easily redisperse any precipitate by shaking. The jet-only-deposition (JOD) experiments of silver in ethanol produced samples which had a much fainter, but otherwise similar pink hue. However, jet-only-deposition experiments did not produce any precipitate.

**Fig. 7:**
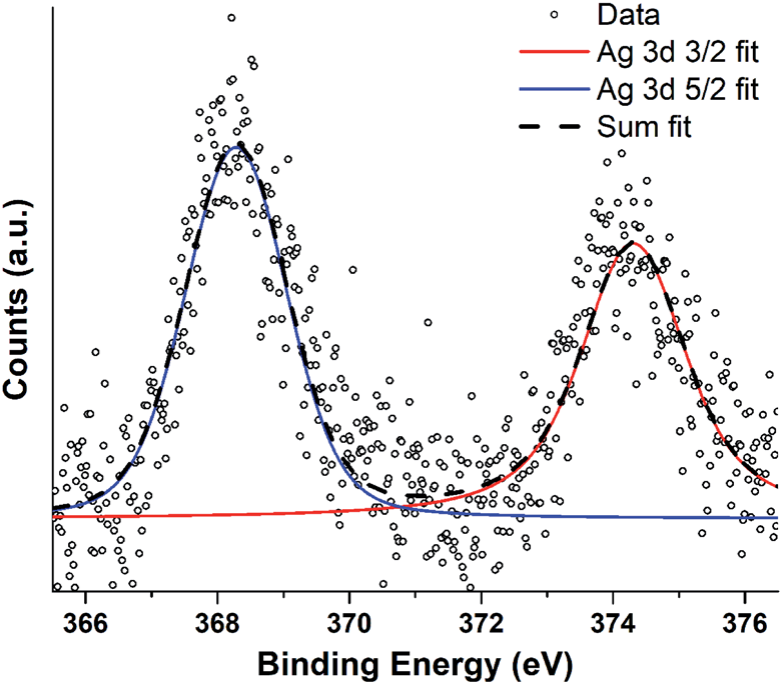
XPS spectra recorded from JCD sample of silver deposited in ethanol drop-cast onto freshly cleaved HOPG surface. Spectra were recorded with an excitation energy of 1253.6 eV (Mg Kα) and an analyser constant acceptance energy of 20 eV.

This pinkish colour is reflected in the UV-visible absorption spectra (Fig. 7), where the JOD and JCD samples, both silver in ethanol, show a long, low absorption from 400 to 600 nm, with a peak at 450 nm (Fig. 9). Sizes from TEM (Fig. 3) show that the silver colloid has a broad particle size distribution. Moreover there is evidence for spherical (Fig. 3), and faceted particles (Fig. 4A and B). Whilst high quality data relating nanoparticle size to peak position exists,^63^ the peak position can be substantially shifted by different particle morphologies,^65,66^ surface oxide^67,68^ and surface chemistry.^64,69^ We ascribe the observed plasmon resonance in silver–ethanol samples to the contributions of many resonances of different particle sizes and morphologies.

**Fig. 8:**
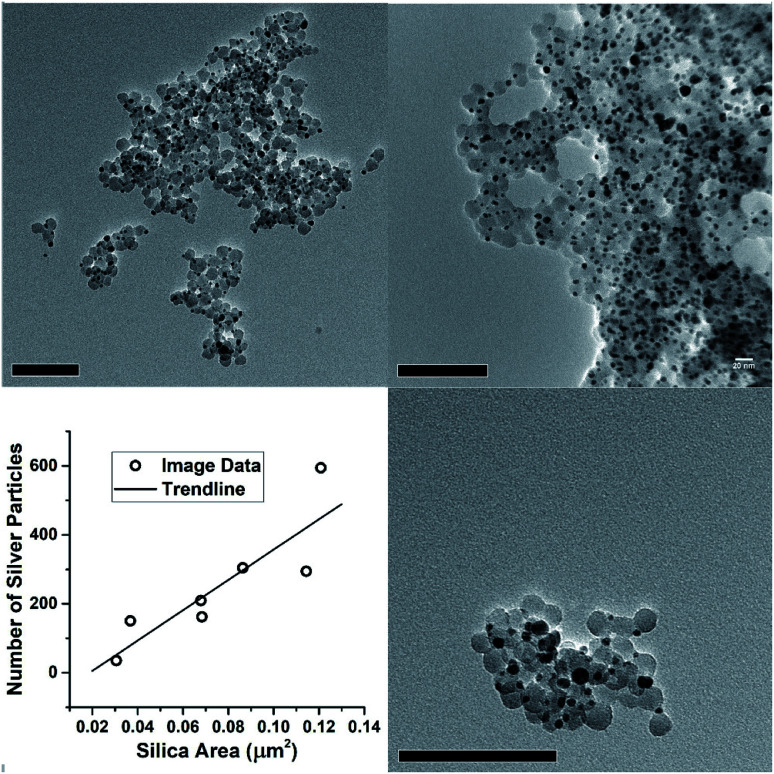
Transmission electron microscopy survey images of silver decorated LUDOX particles, and the relationship between particle counts and total image area filled with silica nanoparticles. The images are a subset selected for clear contrast between substrate and silica particles and absence of substrate features such as holes, webbing or folds. Scale bars are 100 nm.

**Fig. 9:**
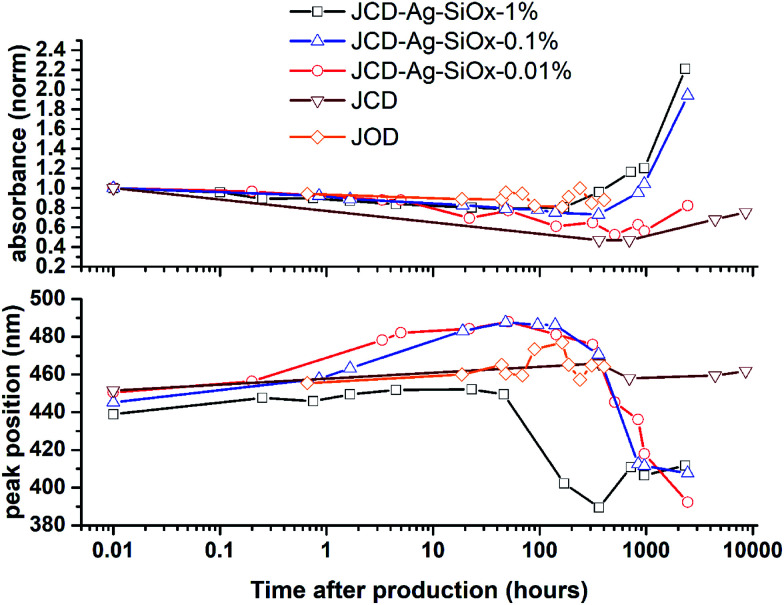
Evolution of optical absorption spectra over time, showing the peak absorbance normalised to the *t* = 0 spectra, and the peak position.

From a simple comparison of the relative cross sections of the cold trap entrance and the liquid jet we could assume that substantially more metal atoms would be deposited into solvent in the JCD regime. An estimate of the total loading of the solvent during sample production, in concentrations of mg L^−1^, is shown in column one of Table 1. These do not agree with concentrations measured by AAS or inferred from UV-vis, which only measured un-precipitated material. However, these do provide a good estimate of the ratio of concentration between samples produced by the JCD and JOD methods, of roughly 16 : 1. We can also estimate that, in a JCD sample, approximately 1/15 of the silver will be mixed into the jet before it freezes, meanwhile the remainder will be deposited into the ice.

We also noticed that both absorption spectra for JCD and JOD both show a strong absorption ‘shoulder’ between 270 nm and 300 nm. Absorption in this region has been observed for aqueous solutions of Ag_2_ and Ag_3_ clusters;^70^ and for small (less than 1 nm) silver clusters in ion exchanged glass.^71^ We suggest that a proportion of the total silver is in the form of dissolved atomic silver and small silver clusters.

Silver nanoparticle solutions produced by Jet-Co-Deposition (JCD) were drop cast onto freshly cleaved highly oriented pyrolytic graphite (HOPG) substrates and analysed by XPS. Spectra showed peaks corresponding to metallic silver: Ag3d_3/2_ at 374.3 pm 0.3 eV and Ag3d_5/2_ at 368.3 pm 0.3 eV. Samples produced by Jet-Only-Deposition (JOD) were not recorded due to the similarities observed in HRTEM lattice planes and fringes, indicating that metallic silver was present in both samples (Fig. 4), coupled with the low XPS signal in JOD samples. JCD-SiO_*x*_ were analysed, however, the signal from silicon was so highly shifted by charging (despite efforts at neutralisation by electron flooding) that silver peaks could not be assigned to either metallic or oxide silver with any confidence.

HRTEM and EDX measurements (Fig. 4) of JCD and JOD samples show that the particles are predominantly silver in composition. Atomic resolution images show that pure silver particles are present. We assume that a small amount of solvent (either ethanol or water contamination) is present on the silver nanoparticle surface based on the small oxygen signal observable in the EDX survey spectrum (0.525 keV) and mapping (Fig. 4, second from left, blue). Taking into account the atomic resolution images, measurements of lattice spacings from FFTs of images, and the XPS measurements of JOD samples, we assess the majority of nanoparticles as pure metallic silver, with metallic silver surfaces.

We can see that the JCD and JOD samples have similar properties. Both have broad size distributions (Fig. 3), with the JOD samples having a higher proportion of smaller particles in the total distribution. The smaller sizes of the JOD sample correlate with the lower loading and hence lower silver concentration achieved during the JOD experiment (Table 1). It is plausible that lower silver concentration combined with higher mobility of Ag within the liquid jet (as opposed to in the ice ‘slush’ in JCD) will result in smaller clusters. However, we also suspect that depositing directly onto the liquid jet also avoids the growth of larger agglomerates on the ice surface. During a JCD experiment, the liquid jet does not necessarily cover the cold trap internal surface evenly, or ‘refresh’ the coverage of the same spots, possibly allowing partial films or regions of high silver concentration to build up in the frozen sample. By blocking the deposition into the cold trap, the JOD experiment avoids this.

Samples of JCD-SiO_*x*_ showed essentially a similar colour to the JCD and JOD samples immediately after production (N.B. to reiterate, JCD-SiO_*x*_ was prepared in methanol, both JCD and JOD were prepared in ethanol). This is visible in the UV-vis spectra recorded immediately after production (Fig. 6, solid line), showing a broad absorption peak centred at 450 nm. All three concentrations of JCD-SiO_*x*_ show similar aging behaviour over time (Fig. 6 and 9). The plasmon resonance intensity initially drops relative to the initial measurement, then the peak narrows and intensifies. The time over which the peak is falling, the total amount the peak falls and the final peak intensity are all correlated with the concentration of silica in the sample. The more silica, the shorter the duration of the falling intensity, the less intensity is initially lost and the greater the final intensity. Also, samples with lower silica concentration also have qualitatively higher observable precipitate. This is also illustrated in the silver concentrations recorded in Table 1, where the measured silver concentration by AAS is much higher than either JCD or JOD, and also much closer to the total estimate of silver deposited into the sample.

Sputtering silver into pure methanol produced the same pinkish colour, which immediately agglomerated into large, optically inactive particles – substantial agglomeration was observable before a UV-visible spectrum could be recorded. After 3 hours the silver had completely agglomerated and the solvent was clear. Agglomerates could not be redispersed by shaking or sonication.

These results are consistent with the LUDOX solution initially stabilising the silver. This is likely to be from the alkaline LUDOX solution. The original solution has a pH of 9.7, where acidic silanol groups on the particle surface are stabilised by sodium counter-ions. Essentially, the excess OH^−^ ions can stabilise positively charged silver atoms and clusters. Calculations of the proportion of silver relative to silica in as-produced solutions show considerable variation between samples (Table 2). Consequently, those samples with more colloidal silica also show lower immediate agglomeration and precipitation of silver (both observed precipitate and measured UV-visible absorption).

**Table 2:** Percentages of silver in silver/silica composites. Samples are labeled as JCD-SiO_*x*_ suffixed with the mass percentage of silica in the original solution. The percentages of silver listed are the molar percentages of silver in the total silver/silica composite

Sample	Molar% Ag
JCD-SiO_*x*_ 0.01%	10.0 ± 1.9%
JCD-SiO_*x*_ 0.1%	3.3 ± 1.4%
JCD-SiO_*x*_ 1%	0.4 ± 0.3%

However, the next phase of the reaction shows that the distribution of silver nanoparticles begins to be substantially altered. This is most visible in the dramatic change of nanoparticle absorption peak position and width, best illustrated in Fig. 6. The peak absorbance shifts to around 410 nm, and narrows substantially. Interestingly, these behaviours and spectra match a digestive ripening process, similar to that observed by Smetana *et al.* after production of silver nanoparticles in 2-butanone by the SMAD method, followed by reflux in 4-*tert*-butyl-toluene.^27^

Microscopy of the JCD-SiO_*x*_ samples also shows the narrowing of the size distribution that would be expected from a digestive ripening process (Fig. 3). The narrowing of the size distribution will narrow the inhomogeneously broadened plasmon resonance spectrum, as the contribution to the absorbance will come from a smaller sub-set of particles. Moreover, as the digestive ripening process can involve the differential re-dissolution and re-deposition of metal from one particle size or morphology to another, this can select a favoured crystal morphology, potentially further narrowing the optical absorption. The proximity or attachment of plasmonic nanoparticles to surfaces can also modify the peak position of the plasmon resonance relative to the equivalent free particle.^72–75^

However, the microscopy also shows that the silver nanoparticles are ‘attached’ to the silica spheres (Fig. 3c, 4C and 8). Of the approximately 2000 silver particles analysed for the size distribution (Fig. 3d and e) less than 1% were unattached to the silica particles. This was tested by shaking the JCD-SiO_*x*_ sample prior to microscopy (Fig. 4D); in this case nanoparticles were dispersed throughout the sample as well as on the silica. However, when unshaken, and allowed to settle (Fig. 4C), the colloid showed that practically all silver nanoparticles were attached to silica spheres. This shows that unattached silver nanoparticles do not preferentially attach to the silica particles as they are drying on the TEM grid, providing strong evidence that the particles attached in TEM images are also attached in solution. The imagery also shows that the distribution of silver on silica is even (Fig. 8) – with approximate proportionality between the number of silver particles and the total area covered by silica.

**Fig. 10:**
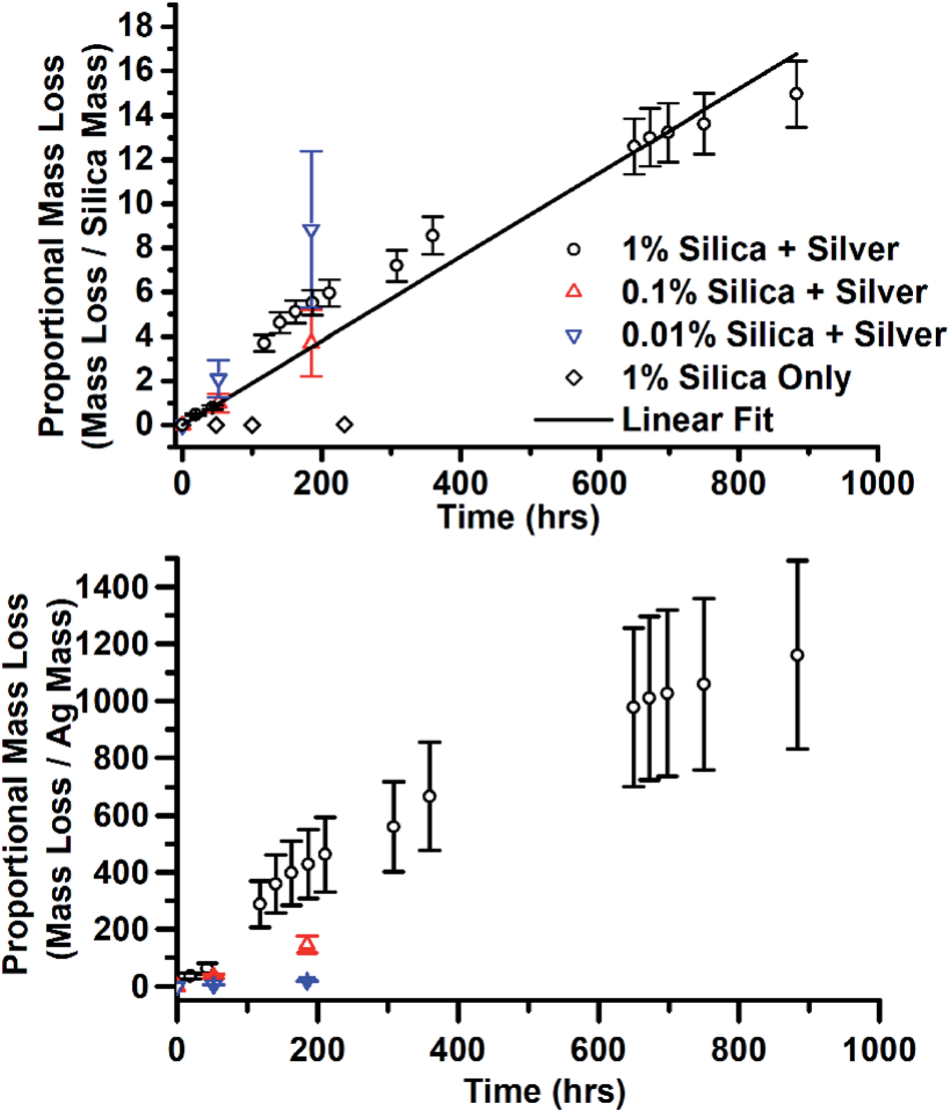
Mass loss from JCD-SiO_*x*_ samples, normalised by the mass of both silica and silver in the sample. The line is a linear least squares regression to all data points from all samples, taking into account errors.

There was another effect of the addition of silica and the variation of silica concentration. In the sample with the highest concentration of silica, JCD-SiO_*x*_-1%, after the final, 90 day measurement, it was observed that a substantial quantity of the solvent had disappeared from the sample vial. Over the same time, the prepared stock LUDOX-methanol solutions, without deposited silver, had lost no solvent. The remaining solution was split – one half was rediluted to as near as possible the original concentration. The other half was allowed to dry over time. Once it was found that the 1% silica sample consistently lost solvent, controlling for the sample vial used, the mass of all three JCD-SiO_*x*_ samples was measured over time. All three samples lost mass over time. The mass lost from the solutions is shown in Fig. 10, normalised by the mass of silica or silver in the solution. The stronger proportionality shown by the normalisation by silica mass strongly suggests that it is the combined silica–silver material rather than the silver alone which has catalytic properties. The linear fit to data in Fig. 10 corresponds to a rate of 1.63 × 10^−7^ mol g^−1^ s^−1^, in mols methanol evolved away each second per gram of catalyst in the solution.

The reaction by which methanol was removed from these samples is unknown. The silver–silica system is a known catalyst for a variety of methanol reactions^76–78^ as well as other industrially relevant reactions.^79–81^ The most likely reaction here is the oxidation of methanol – either partial oxidation of methanol to CO_2_ and H_2_, or the full oxidation to CO_2_ and H_2_O. Although the reaction is not well characterised we have been able to largely rule out the production of methane or formaldehyde by transmission FTIR of a gas cell connected to a reservoir of methanol and catalyst.

During solvent preparation, it was determined that concentrations of LUDOX greater than 5% in methanol rapidly gelled on any agitation. Similarly, as JCD-SiO_*x*_ samples lost mass, they gelled and then rapidly dried out. After drying, the material lost colloidal stability and could no longer by redispersed in methanol.

The measurements of reaction rate in Fig. 10 of the 1% sample was taken from a sample where all of the methanol (and a top-up) had already been evolved away, the sample dried into a crust, then crushed to powder. This meant it had already evolved away approximately 150 times (including the top-up) its own mass with minimal reduction in catalytic rate.

The even dispersion of silver onto silica by this method suggests that it could find practical applications in catalyst synthesis: the even loading of catalytically active particles on supports is key for practical applications of catalysts and is often inadequate in currently used industrial catalysts.^78^

## 4 Conclusions

We have shown that it is possible to inject a liquid jet of ethanol or methanol into vacuum whilst simultaneously DC sputtering silver. The sputtered silver atoms can be captured in the liquid jet, or captured in the cold trap along with the liquid jet. Mixing silver with ethanol, by either of these methods, produced a pink nanoparticle solution with a broad particle size distribution and range of morphologies. When depositing on the jet only, the overall nanoparticle size distribution leans towards smaller particles, whilst depositing into the cold trap produced larger particles. This is likely due to both the increased concentration of silver and the potential reduction of mobility when depositing into the cold trap.

Sputtering silver with methanol produced a pink solution which immediately agglomerated. However, when solutions of 0.01%, 0.1% and 1% LUDOX-HS colloidal silica in methanol were used as solvents, sputtering silver produced stable colloids. The proportion of silver stabilised was proportional to the concentration of silica in solution. Additionally, the silver–silica solution ripened over a period of months; the particle size distribution narrows; the plasmon resonance absorption narrows; and the plasmon peak blue-shifts. Finally, the silver–silica system shows probable catalytic properties for methanol oxidation.

In summary, we have demonstrated for the first time atomic vapour deposition onto or alongside liquid jets of alcohols. This provides a method to mix metal atoms directly with common laboratory solvents to enable new reactions, synthesis processes, or analytical goals. We have directly illustrated the synthesis of polydisperse silver nanoparticles, the ripening of silver nanoparticles on silica particles, and finally the demonstration of a functional system. This demonstration suggests an immediate application: improving the dispersion of metal particles on supporting oxide particles for heterogenous catalysis.

## Conflicts of interest

There are no conflicts of interest to declare.

## Supplementary Material
